# A Novel Scoring System for Pilonidal Disease: Predicting Complications and Guiding Surgical Management

**DOI:** 10.7759/cureus.95454

**Published:** 2025-10-26

**Authors:** Ahmed H. Amer, Emma Camilleri, Fouad Ashoush, Ahmed Abdelrahim, Mohamed Arakib, Ibtehal Elgaabari, Mohamed Abdelaal, Sameh ELabd, Mohamed Abdelhalim

**Affiliations:** 1 General Surgery, Tanta University, Tanta, EGY; 2 Surgery, County Durham and Darlington National Health Service (NHS) Foundation Trust, Durham, GBR; 3 General Surgery, Northumbria Healthcare National Health Service (NHS) Foundation Trust, North Tyneside, GBR; 4 General Surgery, Health Education England North East, Newcastle, GBR; 5 General Surgery, Tanta University Hospitals, Tanta, EGY; 6 General Surgery, Chelsea and Westminster Hospital National Health Service (NHS) Foundation Trust, London, GBR; 7 General and Colorectal Surgery, County Durham and Darlington National Health Service (NHS) Foundation Trust, Darlington, GBR

**Keywords:** local excision, midline pits, pilonidal sinus, recurrence, scoring system, wound dehisence

## Abstract

Background: Pilonidal disease is a chronic condition of the sacrococcygeal region, frequently affecting young adults and associated with high rates of recurrence and postoperative complications. Numerous surgical techniques have been described, yet there is no universally accepted classification or staging system to guide treatment decisions. This lack of standardization contributes to inconsistent outcomes and difficulty in predicting prognosis.

Objective: This study aimed to develop and validate a novel preoperative scoring system, the Hazem-Talaat score, to assess disease severity, predict recurrence, and facilitate surgical planning in pilonidal disease.

Methods: A prospective study was carried out on 156 patients (108 males, 48 females) aged 18-45 years admitted with primary or recurrent pilonidal disease to the General Surgery Department at Tanta University, Egypt, between June 2023 and June 2024. Patients with recurrent disease after flap repair or with hematological disorders were excluded, as the reason for this is to avoid anatomical distortions caused by flap surgeries. Preoperative variables, including BMI, sex, hair distribution, diabetes mellitus, number and location of pits, distance from the anus, history of abscess, and prior recurrence, were incorporated into the Hazem-Talaat score. All patients underwent standardized asymmetrical elliptical excision with off-midline closure and were followed for at least six months to assess wound complications and recurrence.

Results: The mean age of participants was 27.3 ± 6.5 years, and the mean BMI was 29.1 ± 4.6 kg/m². Recurrence occurred in 19 patients (12.2%), while wound dehiscence was noted in 26 patients (16.7%). Recurrence was significantly associated with male gender (p = 0.010), higher BMI (p < 0.001), diabetes mellitus (p < 0.001), prolonged operative time (p < 0.001), prior recurrence (p < 0.001), more than 10 midline pits (p < 0.001), lateral extension (p < 0.001), hairy back (p < 0.001), distance from anus < 5 cm (p < 0.001), and previous abscess formation (p < 0.001). Patients who developed recurrence had significantly higher Hazem-Talaat scores (mean 22.1 ± 4.5) compared with those without recurrence (mean 14.0 ± 3.1; p < 0.001). A cut-off value of 21 was determined using a receiver operating characteristic (ROC) curve.

Conclusion: The Hazem-Talaat score provides an objective, multifactorial tool that integrates clinical and anatomical factors to predict recurrence and stratify patients with pilonidal disease. Its application in surgical planning may improve outcomes by tailoring the choice of operative technique to disease severity. Further multicenter studies with longer follow-up are warranted to externally validate this system.

## Introduction

Pilonidal disease is a chronic condition that presents in various ways. It usually presents with single or multiple discharging sinuses that are occasionally painful in the region of the natal cleft [1]. Many procedures have been described for its treatment; however, a widely accepted and standard approach has not yet been agreed upon by surgeons. The most common procedures include excision with either primary closure or left open for healing, and excision with flap reconstruction (rhomboid excision and Limberg flap). All procedures had a considerable rate of complications, including infection, wound dehiscence, and recurrence [2,3]. Some authors have suggested that the lack of a universally accepted staging or scoring system contributes to the absence of a standard surgical approach. It is unreasonable to treat a simple midline single-pit disease in the same way as an extensive multi-pit disease. Although trials have attempted to establish staging systems, none have yet gained widespread acceptance [4,5].

Beal et al. identified eight scoring or staging systems in the literature; however, none were considered sufficiently structured to reliably predict prognosis or to correlate disease extent with treatment options [6]. Three studies suggested that a single-pit disease within the intergluteal sulcus should be classified as a distinct category, representing a mild form that could be managed differently from multiple-pit (extensive) disease [7-9]. Several randomized controlled studies suggest superiority of a flap repair over excision with primary closure to reduce wound complications and recurrence. However, few of these studies have reported the incidence of postoperative numbness, which may be as high as 19% following the Limberg flap. Presently, no data has been reported regarding patient satisfaction rates. This is a vital component to consider, especially when scars can cause considerable cosmetic disfigurement, particularly with extensive resection. This may have a significant psychological impact on patients, potentially affecting self-esteem, body image, and overall quality of life, particularly in the young population [10].

To the best of our knowledge, none of the available studies has succeeded in establishing a universally accepted classification or staging system for the disease, largely due to the heterogeneity of clinical presentations, the diversity of surgical techniques, and in some cases, the limited number of patients included. In a previous study conducted by the authors, similar limitations were encountered, and the authors therefore sought to establish a novel scoring system for pilonidal disease. Our findings indicated that both the number of midline pits and the presence of certain factors (e.g., diabetes) were associated with an increased risk of wound complications and disease recurrence [11].

## Materials and methods

This prospective study was conducted on 156 adult patients admitted to the General Surgery Department at Tanta University in Egypt. The study was carried out over 13 months (June 2023-June 2024) after obtaining ethical committee approval and informed written consent. This study was reviewed and approved by the Research Ethics Committee of Tanta University, Faculty of Medicine, under approval number 36264PR221/6/23. All patient information used in this study/paper has been anonymized to protect individual privacy. No identifiable personal data is included. The study complies with ethical standards concerning the confidentiality of patient records, in accordance with the Declaration of Helsinki.

The study included adult patients aged 18-45 years with simple or complex (multiple pits) pilonidal disease. Patients with recurrent disease after previous flap repair and patients with hematological disorders were excluded. The preoperative patient characteristics were established. Each characteristic was assigned a score based on the severity of the disease (Table 1).

**Table 1:** Hazem-Talaat score for the assessment of the disease pre-operatively

Patients' characteristics	Score
Weight	
- BMI >30	2
- BMI<30	1
Sex	
- Male	2
- Female	1
Hair character	
- Hairy back with hard-textured hair	2
- Non-hairy back	1
Diabetes Mellitus	
- Yes	3
- No	1
Characteristics of the disease	
Previous surgery (recurrence)	
- Yes	3
- No	1
Number of midline pits	
- 0-5	2
- 5-10	4
- >10	6
Presence of pits lateral to the midline	
- Yes	3
- No	1
Distance of the nearest pit to the anus	
- > 5 cm	1
- < 5 cm	3
Previous abscess formation	
- Present	3
- Not	1

Operative technique

After field preparation with wide strapping and sterilization, an asymmetrical elliptical incision was made to include all visible pits, with the two angles displaced approximately 2 cm off the midline toward the side of any additional pits. If all pits were confined to the midline, either side was chosen. Excision was carried down to the sacrococcygeal fascia. Lateral pits distant from the main incision were excised separately. Following complete excision, layered closure was performed with an effort to displace the skin suture line away from the midline. A drain was inserted and typically removed after approximately one week.

Post-operative care

Patients were supplied with pain control medications and were instructed in self-hygiene, wound care, dressing, and how to carefully deal with the drain. Follow up for wound complications and possible recurrence for at least six months. Any unexpected risk that would appear during the procedure was announced to the participants and the ethical committee at the time, and adequate measures would be taken.

Statistical analysis of the data

Data were entered into a secure password-protected computer and analyzed using SPSS Software, version 20.0. (IBM Corp, Armonk, NY). Categorical data was represented as numbers and percentages. Chi-square test was applied to compare the two groups. Alternatively, Fisher's Exact and Monte Carlo corrections were applied when more than 20% of the cells had expected counts less than 5. Continuous data was tested for normality by the Shapiro-Wilk test. Quantitative data were expressed as range (minimum and maximum), mean, standard deviation, median, and interquartile range (IQR). Student's t-test was used to compare two groups for normally distributed quantitative variables. On the other hand. The Mann-Whitney test was used to compare two groups for not normally distributed quantitative variables. The receiver operating characteristic curve (ROC) was used to determine the diagnostic performance of the markers. An area of more than 50% gives acceptable performance, and an area of about 100% is the best performance for the test. Significance of the obtained results was judged at the 5% level.

## Results

This study included 108 males (69.2%) and 48 females (30.8%). Participants’ ages ranged from 18 to 45 years, with a mean (± SD) age of 27.31 (± 6.48) years. Body mass index (BMI) ranged from 19.0 to 41.0 kg/m², with a mean (± SD) of 29.12 (± 4.63) kg/m². Operative time ranged from 21.0 to 75.0 minutes, with a mean (± SD) duration of 44.42 (± 12.63) minutes. Twenty-one patients (13.5%) had diabetes mellitus, and 26 (16.7%) presented with recurrent pilonidal sinus. Direct postoperative complications, including bleeding and infection, were observed in three patients (1.9%). The number of pits was fewer than 5 in 83 patients (53.2%), between 5 and 10 in 50 patients (32.1%), and more than 10 in 23 patients (14.7%). Lateral pits were present in 42 patients (26.9%). Regarding hospitalization, 154 patients (98.7%) stayed for one day, one patient (0.6%) stayed for two days, and another patient (0.6%) stayed for three days. The length of hospital stay ranged from one to three days, with a mean (± SD) of 1.02 (± 0.18) days. Drain removal occurred between 7 and 14 days postoperatively, with a mean (± SD) of 7.39 (± 1.34) days. Wound dehiscence was observed in 26 patients (16.7%), and recurrence occurred in 19 patients (12.2%). A hairy back was noted in 64 patients (41.0%), and the distance from the anus was less than 5 cm in 38 patients (24.4%). Previous abscess history was reported in 49 patients (31.4%). The difficulty score ranged from 10.0 to 27.0, with a mean (± SD) of 14.96 (± 4.21) (Table 2).

**Table 2:** Distribution of the studied cases according to different parameters (n = 156) SD: Standard deviation, IQR: Interquartile range.

Parameters	No. (%)
Gender	
Male	108 (69.2%)
Female	48 (30.8%)
Age (years)	
Min. – Max.	18.0 – 45.0
Mean ± SD.	27.31 ± 6.48
Median (IQR)	26.0 (22.0 – 31.50)
BMI (kg/m^2^)	
Min. – Max.	19.0 – 41.0
Mean ± SD.	29.12 ± 4.63
Median (IQR)	29.0 (26.0 – 32.0)
Operative time (min)	
Min. – Max.	21.0 – 75.0
Mean ± SD.	44.42 ± 12.63
Median (IQR)	45.0 (34.0 – 52.50)
Ddiabetes mellitus	21 (13.5%)
Recurrent pilonidal sinus	26 (16.7%)
Direct post-operative complication: bleeding, infection	3 (1.9%)
Number of pits	
Less than 5	83 (53.2%)
5 – 10	50 (32.1%)
More than 10	23 (14.7%)
Presence of lateral pits	42 (26.9%)
Hospital stay (days)	
1	154 (98.7)
2	1 (0.6)
3	1 (0.6)
Mean ± SD.	1.02 ± 0.18
Drain removal (days)	
Min. – Max.	7.0 – 14.0
Mean ± SD.	7.39 ± 1.34
Median (IQR)	7.0 (7.0 – 7.0)
Wound dehiscence	26 (16.7%)
Recurrence	19 (12.2%)
Hairy back	64 (41.0%)
Distance from the anus less than 5 cm	38 (24.4%)
Previous abscess	49 (31.4%)
Score	
Min. – Max.	10.0 – 27.0
Mean ± SD.	14.96 ± 4.21
Median (IQR)	14.0 (12.0 – 17.0)

Out of 108 male patients, 18 experienced recurrence, indicating a significant association between male gender and recurrence (P = 0.010). There was no significant difference in age between the recurrence and non-recurrence groups. Higher BMI was significantly correlated with recurrence, as was prolonged operative time, which may be attributed to increased tissue manipulation or the use of diathermy for hemostasis. Recurrence was also significantly associated with a history of recurrent disease; 11 of the 26 patients with previous recurrence experienced another episode. Additionally, diabetes mellitus showed a significant relationship with recurrence, with 10 of 21 diabetic patients developing recurrent disease. Among the 23 patients with more than 10 pits, 12 had recurrence, a statistically significant finding. A hairy back was noted as a factor, and the distance from the anus was inversely related to recurrence; the shorter the distance from the anal verge, the higher the likelihood of pilonidal sinus recurrence. Furthermore, a history of previous pilonidal abscess prior to surgery was significantly associated with recurrence. Patients classified as high-risk according to the new Hazem-Talaat scoring system experienced a significantly higher rate of disease recurrence (p < 0.05), supporting the scoring model’s predictive validity (Table 3).

**Table 3:** Comparison between not recurrent and recurrent according to different parameters (n = 156) IQR: Interquartile range, SD: Standard deviation, t: Student t-test, U: Mann-Whitney test, χ2: Chi-square test, FET: Fisher Exact test, MC: Monte Carlo test, p: p-value for comparing between the two studied groups. *: Statistically significant at p ≤ 0.05.

		Not recurrent (n = 137)			Recurrent (n = 19)		Test of Sig.	p
Gender								
Male		90 (65.7%)			18 (94.7%)		χ^2^= 6.607^*^	0.010^*^
Female		47 (34.3%)			1 (5.3%)			
Age (years)								
Min. – Max.		18.0 – 45.0			19.0 – 40.0		χ^2^= 0.114	0.909
Mean ± SD.		27.29 ± 6.44			27.47 ± 6.88			
Median (IQR)		26.0 (22.0 – 31.0)			26.0 (21.50 – 31.50)			
BMI (kg/m^2^)								
Min. – Max.		19.0 – 41.0			21.0 – 40.0		χ^2^= 3.844	<0.001^*^
Mean ± SD.		28.61 ± 4.43			32.79 ± 4.52			
Median (IQR)		29.0 (26.0 – 31.0)			34.0 (30.0 – 35.0)			
Operative time (min)								
Min. – Max.		21.0 – 75.0			26.0 – 74.0		U= 632.500	<0.001^*^
Mean ± SD.		42.91 ± 11.77			55.32 ± 13.59			
Median (IQR)		44.0 (33.0 – 50.0)			61.0 (43.50 – 66.0)			
Diabetes mellitus		11 (8.0%)			10 (52.6%)		χ^2^=28.495	^FE^p<0.001^*^
Recurrent pilonidal sinus		15 (10.9%)			11 (57.9%)		χ^2^=26.477	^FE^p<0.001^*^
Direct post-operative infection		0 (0.0%)			3 (15.8%)		χ^2^=22.056	^FE^p=0.002^*^
Number of pits								
Less than 5		79 (57.7%)			4 (21.1%)		χ^2^= 40.384	<0.001^*^
5 – 10		47 (34.3%)			3 (15.8%)			
More than 10		11 (8.0%)			12 (63.2%)			
Presence of lateral pits		27 (19.7%)			15 (78.9%)		χ^2^=29.762	<0.001^*^
Hairy back		48 (35.0%)			16 (84.2%)		χ^2^=16.676	<0.001^*^
Distance from anus less than 5 cm		23 (16.8%)			15 (78.9%)		χ^2^=34.990	^FE^p<0.001^*^
Previous abscess		35 (25.5%)			14 (73.7%)		χ^2^=17.946	<0.001^*^
Score								
Min. – Max.		10.0 – 25.0			12.0 – 27.0		U= 229.500	<0.001^*^
Mean ± SD.		13.96 ± 3.07			22.11 ± 4.48			
Median (IQR)		13.0 (12.0 – 16.0)			23.0 (20.50 – 25.0)			

**Figure 1:**
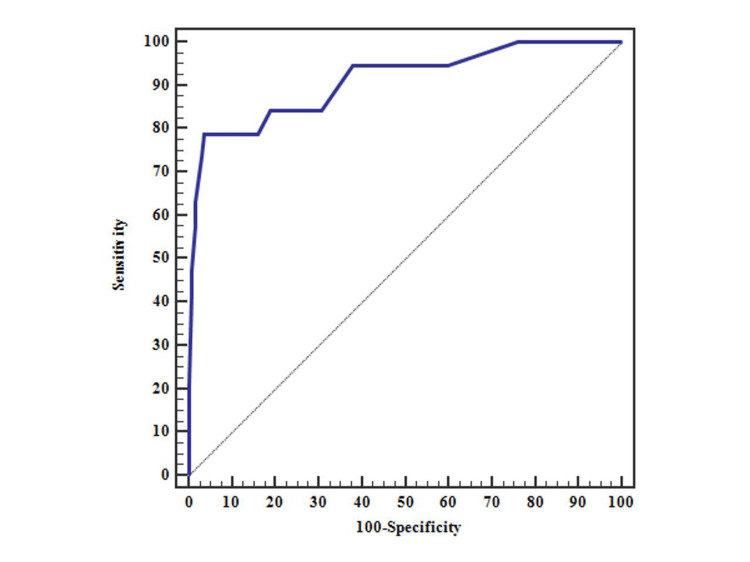
ROC curve for score to predict recurrent (n= 19) from not recurrent (n = 137) ROC: Receiver operating charecteristic

Receiver operating characteristic (ROC) curve analysis was performed to evaluate the predictive performance of the proposed scoring system in assessing the risk of pilonidal sinus recurrence. The analysis demonstrated an area under the curve (AUC) of 0.912, indicating excellent discriminatory ability. A cut-off score of ≥21 was identified as the optimal threshold for predicting recurrence. At this cut-off, the sensitivity was 63.16%, and the specificity was 98.54%, suggesting that the scoring system is highly specific in identifying patients at risk for recurrence. Patients scoring 21 or higher may therefore be considered high-risk and could benefit from more advanced or alternative treatment strategies (Figure 1) (Table 4).

**Table 4:** Prognostic performance for score to predict recurrent (n= 19) from not recurrent (n = 137) AUC: Area under a curve, p-value: Probability value, CI: Confidence intervals, NPV: Negative predictive value, PPV: Positive predictive value. *: Statistically significant at p ≤ 0.05.

	AUC	p	95% C.I	Cut off	Sensitivity	Specificity	PPV	NPV
Score	0.912	<0.001^*^	0.831 – 0.993	≥21	63.16	98.54	85.7	95.1

## Discussion

Pilonidal disease is a chronic condition characterized by the formation of sinus tracts in the sacrococcygeal region and, less commonly, in other areas such as the groin, umbilicus, or axilla. The high recurrence rates of pilonidal disease and the lack of consensus on the most effective surgical approach make it a difficult condition for patients and surgeons alike [12]. No single surgical technique has been shown to be universally superior, and recurrence rates still range from 5% to 30% in the literature. These techniques range from basic excision with secondary healing to more complex flap procedures, such as the Karydakis, Limberg, or Bascom techniques [13,14]. The complex interplay between patient- and disease-specific variables, such as obesity, diabetes, and hair density, as well as disease-specific variables like sinus size, number, and lateral extension, is a major contributor to the unpredictability of surgical results [15].

The lack of a standardized, easy-to-use scoring system for assessing the severity of pilonidal disease has hindered effective communication among clinicians and research efforts. Our study highlights the significance of multiple clinical and anatomical factors in predicting outcomes after surgical management of pilonidal disease. The novel scoring system we developed successfully integrated these parameters into a single index, demonstrating clear discrimination between recurrent and non-recurrent cases. Patients who experienced recurrence had consistently higher scores, confirming the validity of this tool as a predictor of complications and surgical failure.

The recurrent group exhibited a substantially elevated body mass index (BMI), which was one of the strongest predictors. The natal cleft is known to be deepened, friction and perspiration are increased, and wound healing is impaired by obesity, all of which contribute to recurrence [16]. Similarly, diabetes mellitus was identified as a significant risk factor, which is consistent with the established evidence that hyperglycemia inhibits tissue perfusion, leukocyte activity, and collagen synthesis, thereby increasing the likelihood of infection and dehiscence [17].

These sinus tracts were equally critical in terms of their geometry and burden. There was a significantly increased risk of recurrence among patients with more than 10 fissures or lateral extensions. These characteristics are indicative of a more intricate disease, which increases the probability of residual tracts following excision. Previous reports have underscored the likelihood of failure in the event that multiple or off-midline fissures are not adequately excised [18,19]. Furthermore, recurrence was strongly associated with a short distance from the anus (<5 cm). This is likely due to the fact that this location is associated with a more contaminated, warmer, and deeper fissure environment, which complicates the healing process [20].

Hair biology was also a factor. In recurrent patients, the presence of a hairy back was substantially more prevalent, which is consistent with the well-established pathogenesis of pilonidal sinus as a foreign-body reaction to shed hairs penetrating the midline skin [21]. Similarly, a history of previous abscesses was associated with recurrence, likely as a result of chronic inflammation, fibrosis, and distorted tissue planes that complicate subsequent surgery [22].

These observations were corroborated by postoperative and operational findings. In severe instances, technical difficulty and persistent dead space were reflected in the association between recurrence and prolonged operative time and delayed drain removal. Additionally, the recurrent group exhibited substantially higher rates of wound dehiscence and early complications, including infection, which further supports the notion that the early breakdown of closure is a strong predictor of long-term failure [23,24].

In conjunction, these results emphasize the multifactorial nature of pilonidal disease and substantiate the necessity of an integrated risk assessment tool. Recurrent cases scored substantially higher than non-recurrent cases, and the proposed scoring system demonstrated strong predictive ability by capturing all of these determinants. In terms of clinical application, this score could assist surgeons in customizing the selection of surgical modality. Patients with low scores may be treated with a straightforward off-midline closure, whereas those with higher scores may necessitate flap-based procedures to reduce the likelihood of recurrence.

This study has several limitations that should be acknowledged. First, the study was conducted in a single center, which may limit the generalizability of the results to other healthcare systems and populations. The second limitation is that the follow-up period of six months may not be sufficient to capture late recurrences, despite the relatively adequate sample size. Third, the scoring system's applicability to this subgroup of complex cases was restricted by the exclusion of patients with recurrent disease following flap repair.

## Conclusions

In conclusion, the scoring system exhibited robust discriminatory capabilities in this cohort, especially by highlighting a cutoff value upon which categorization of patients with risk of recurrence can be done. The proposed Hazem-Talaat scoring system indicates progression toward a more objective evaluation of pilonidal disease, providing a practicable framework that has the potential to enhance patient outcomes and refine surgical decision-making.

Our recommendation is that future research be conducted to validate this innovative scoring system in larger, multi-center cohorts with more diverse patient populations and an extended follow-up of at least two years. Furthermore, randomized controlled trials comparing score-guided surgical planning with standard practice could prove the system's genuine clinical utility in reducing complications and recurrence. In the long term, integrating the scoring system into preoperative assessment protocols may provide surgeons with a practical, evidence-based tool to stratify patients, individualize surgical approaches, and enhance long-term outcomes in pilonidal disease.
